# Characteristics of gestational diabetes subtypes classified by oral glucose tolerance test values

**DOI:** 10.1111/eci.13628

**Published:** 2021-06-13

**Authors:** Grammata Kotzaeridi, Julia Blätter, Daniel Eppel, Ingo Rosicky, Tina Linder, Franziska Geissler, Evelyn A. Huhn, Irene Hösli, Andrea Tura, Christian S. Göbl

**Affiliations:** ^1^ Department of Obstetrics and Gynecology Medical University of Vienna Vienna Austria; ^2^ Department of Obstetrics and Gynecology University Hospital Basel Basel Switzerland; ^3^ Metabolic Unit CNR Institute of Neuroscience Padova Italy

**Keywords:** early gestation, gestational diabetes mellitus, heterogeneity, pregnancy, subtypes

## Abstract

**Background:**

In clinical practice, gestational diabetes mellitus (GDM) is treated as a homogenous disease but emerging evidence suggests that the diagnosis of GDM possibly comprises different metabolic entities. In this study, we aimed to assess early pregnancy characteristics of gestational diabetes mellitus entities classified according to the presence of fasting and/or post‐load hyperglycaemia in the diagnostic oral glucose tolerance test performed at mid‐gestation.

**Methods:**

In this prospective cohort study, 1087 pregnant women received a broad risk evaluation and laboratory examination at early gestation and were later classified as normal glucose tolerant (NGT), as having isolated fasting hyperglycaemia (GDM‐IFH), isolated post‐load hyperglycaemia (GDM‐IPH) or combined hyperglycaemia (GDM‐CH) according to oral glucose tolerance test results. Participants were followed up until delivery to assess data on pharmacotherapy and pregnancy outcomes.

**Results:**

Women affected by elevated fasting and post‐load glucose concentrations (GDM‐CH) showed adverse metabolic profiles already at beginning of pregnancy including a higher degree of insulin resistance as compared to women with normal glucose tolerance and those with isolated defects (especially GDM‐IPH). The GDM‐IPH subgroup had lower body mass index at early gestation and required glucose‐lowering medications less often (28.9%) as compared to GDM‐IFH (47.8%, *P* = .019) and GDM‐CH (54.5%, *P* = .005). No differences were observed in pregnancy outcome data.

**Conclusions:**

Women with fasting hyperglycaemia, especially those with combined hyperglycaemia, showed an unfavourable metabolic phenotype already at early gestation. Therefore, categorization based on abnormal oral glucose tolerance test values provides a practicable basis for clinical risk stratification.

## 1 INTRODUCTION

In clinical practice, gestational diabetes mellitus (GDM) is regarded and treated as a homogenous disease. However, emerging evidence suggests that the diagnosis of GDM possibly comprises different metabolic entities.^1^, ^2^ A deeper understanding of the phenotypic heterogeneity of the disease is necessary to provide effective and individualized treatment strategies. However, it is not yet well understood how to distinguish possible subtypes of GDM in the clinical setting. One practical approach is to classify GDM entities based on glucose concentrations exceeding the recommended fasting or post‐load thresholds within the diagnostic 75‐g 2‐hour oral glucose tolerance test (OGTT) between 24 and 28 weeks of gestation.^3^, ^4^ Thereby, three subgroups of the disease can be separated: first, GDM patients with elevated fasting glucose but normal glucose concentrations after oral glucose load; second, GDM patients with increased post‐load glucose but normal fasting glucose levels; and third, patients with elevated fasting and elevated post‐load glucose concentrations. This classification is also feasible concerning studies on non‐pregnant humans, indicating that fasting and post‐load hyperglycaemia represent different underlying deterioration processes of glucose metabolism.^5^ However, this concept is less well established in pregnancy and their specific characteristics and risk factors at early gestation are not investigated until now.

Therefore, this study aimed to assess early pregnancy characteristics of GDM entities classified according to the presence of fasting and/or post‐load hyperglycaemia during the diagnostic OGTT. A particular focus was given to the assessment of glucometabolic parameters. The possible requirement of pharmacotherapy in addition to analysis of obstetric outcomes was addressed as a further objective.

## 2 MATERIAL AND METHODS

### 2.1 Study design and participants

In this prospective cohort study, we included a total of 1164 study participants among all women attending the pregnancy outpatient clinic at the Department of Obstetrics and Gynecology, Medical University of Vienna, between January 2016 and July 2019. Women with preconceptionally unrecognized diabetes (diagnosed by HbA1c ≥ 6.5% and/or fasting plasma glucose ≥126 mg/dL at early pregnancy), pregnancy failure or missing OGTT data (e.g. if GDM was diagnosed by elevated fasting glucose levels ≥92 mg/dL and hence the OGTT was not performed) were excluded from this study, resulting in an effective sample size of 1087 cases. More detailed information about included and excluded study participants is provided in the Figure S1. A broad risk evaluation was performed before 16 + 0 weeks of gestation (median gestational age at study entry was 12.9 weeks, IQR 12.3‐13.6), including the assessment of maternal age, parity, obstetric history, family history of diabetes, history of GDM, ethnicity and pregestational body mass index (BMI) by use of self‐reported weight, which has shown a high correlation (*r* = 0.97) with technician‐measured weight in previous validation studies^6^ and is therefore often used in larger epidemiological investigations.^7^ Accordingly, we observed a high correlation between self‐reported and measured weight or BMI of *r* = 0.98 in an independent validation cohort (Figure S2). Weight gain during pregnancy was assessed as difference between body weight at third trimester (last appointment within two weeks before delivery assessed from the patient charts) and pregestational weight. This was available in 428 patients (345 NGT, 33 GDM‐IFH, 35 GDM‐IPH, 15 GDM‐CH). Moreover, a fasting blood examination was performed at the baseline visit to assess fasting plasma glucose (FPG), insulin and C‐peptide, lipids and glycated haemoglobin A1c (HbA1c). The study participants received universal GDM testing by use of a 75‐g 2‐hour OGTT at the late second or early third trimester. Thereby, GDM was diagnosed according to the International Diabetes in Pregnancy Study Groups (IADPSG) recommendations if fasting and/or glucose concentrations after oral glucose load exceeded the proposed cut‐offs (i.e. fasting glucose ≥92 mg/dL; 1 hour post‐load glucose ≥180 mg/dL; 2 hour post‐load glucose ≥153 mg/dL).^3^ In 31 patients with fasting glucose ≥92 mg/dL before 24 weeks of gestation, presence of GDM was verified by early OGTT in accordance with our national guidelines.^8^ Based on the results of the OGTT, study participants were classified as normal glucose tolerant (NGT) if recommended thresholds were not exceeded, as having isolated fasting hyperglycaemia (GDM‐IFH), if fasting glucose was ≥92 mg/dL but 1 and 2 hours post‐load glucose levels were in the normal range, and as having isolated post‐load hyperglycaemia (GDM‐IPH), if 1 and 2 hours glucose concentrations were equal or exceeded 180 and 153 mg/dL, respectively. Patients exceeding both, fasting and post‐load glucose thresholds (either 1 or 2 hours or both), that is combined hyperglycaemia, were classified as GDM‐CH. After diagnosis of GDM all patients received medical nutrition therapy (isocaloric diet containing 40%‐50% carbohydrates, 20% proteins and 30%‐35% fat, divided into three meals and three snacks) and lifestyle advice for 30 minutes and were advised on capillary blood glucose measurement (fasting as well as 1 hour after starting each meal) according to our national guidelines.^8^, ^9^ Glucose‐lowering medication (insulin or metformin) was started if glycaemic targets were not achieved by lifestyle modification (i.e. if fasting or postprandial glucose levels exceeded 95 or 140 mg/dL, according to International Guidelines).^10^ In Austria, the use of metformin in pregnancy (either as single therapy or in combination with insulin) is supported by local guidelines if the patient agrees to this treatment after receiving detailed information about advantages and disadvantages of this therapy.^11^ The Austrian Diabetes Association recommends this strategy especially in insulin‐resistant obese mothers.^9^ Calculations of age‐adjusted and sex‐adjusted percentiles of birth weight were based on international anthropometric standards.^12^ Large for gestational age (LGA) was defined as bodyweight above the 90th percentile. All laboratory parameters, which were assessed at study entry, were measured according to the standard laboratory methods at our certified Department of Medical and Chemical Laboratory Diagnostics (http://www.kimcl.at). Plasma glucose was measured by the hexokinase method with a coefficient of variation (CV) of 1.3%. The levels of insulin and C‐peptide were measured by chemiluminescence immunoassays with CVs of 4%‐7% and 3%‐4%, respectively. HbA1c was assessed by high‐performance liquid chromatography, IFCC standardized and DCCT aligned (CV = 1.8%). Glucose measurements during the diagnostic OGTT were assessed at local public laboratories by use of venous plasma blood samples according to international and local guidelines.^3^, ^8^, ^9^ The study was approved by the Ethics Committee of the Medical University of Vienna and performed in accordance with the Declaration of Helsinki. Written informed consent was obtained from all participants. Reporting of the study conforms to the broad EQUATOR guidelines.^13^

### 2.2 Assessment of insulin sensitivity and β‐cell function

The degree of insulin resistance was assessed by the homeostasis model assessment (HOMA‐IR) and by the quantitative insulin sensitivity check index calculated from insulin (QUICKI‐I) and C‐peptide (QUICKI‐CP).^14^, ^15^ Moreover, we used a modified insulinogenic index from C‐peptide (IGI = FCP [ng/mL]/FPG [mg/dL]), whereby FCP is fasting C‐peptide and FPG is fasting glucose, as an alternative method to estimate insulin secretion.^16^ The product of IGI × FI^−1^, whereby FI is fasting insulin, was used to estimate β‐cell function from fasting parameters (also sometimes called “oral” disposition index (DI)).^17^ To assess the reliability of fasting measurements in order to provide information about insulin sensitivity and β‐cell function, we included a further validation study (n = 45 pregnant women) with dynamic assessment of glucometabolic parameters by use of a 1‐hour frequently sampled intravenous glucose tolerance test (1 hour‐FSIGT) at 13.3 (IQR 12.4‐14.0) weeks of gestation. It was shown that the calculated insulin sensitivity index (CSI, calculated according to Ref.^18^) from FSIGT data was closely associated with insulin sensitivity from fasting parameters (QUICKI‐CP rho = 0.59, *P* < .001 and HOMA‐IR rho = ‐0.52, *P* < .001, respectively). According to β‐cell function, the FSIGT disposition index (calculated as CSI × acute C‐peptide response to glucose) was significantly associated with the disposition index assessed from fasting parameters as well (rho = 0.40, *P* = .007). Details are provided in Figure S2.

### 2.3 Statistical analysis

Continuous variables were summarized by mean ± standard deviation or as median and interquartile ranges (IQR) in case of skewed distribution and compared by analysis of variance or rank‐based inference, respectively. Categorical variables were summarized by counts and percentages and compared by binomial logistic regression. Odds ratios and 95% confidence intervals (95% CI) were additionally calculated for binary outcomes. Tukey's HSD was used for all subgroup (*k* = 4) comparisons to achieve a 95% coverage probability. Associations between metric scaled variables were assessed by Spearman's rho. Statistical analysis was performed with R (version 4.0.2) and contributing packages (especially ‘multcomp’ and ‘nparcomp’).^19^ A two‐sided *P*‐value of ≤.05 was considered statistically significant.

## 3 RESULTS

A total of 194 patients with GDM were identified by the IADPSG recommended thresholds. Thereby, 67 patients showed isolated hyperglycaemia at fasting state and were classified as GDM‐IFH, 83 patients showed isolated increased glucose levels after oral glucose load and were therefore classified as GDM‐IPH, and 44 patients showed combined hyperglycaemia (i.e. elevated fasting and elevated post‐load glucose concentrations, GDM‐CH). 893 women remained normal glucose tolerant.

**FIGURE 1:**
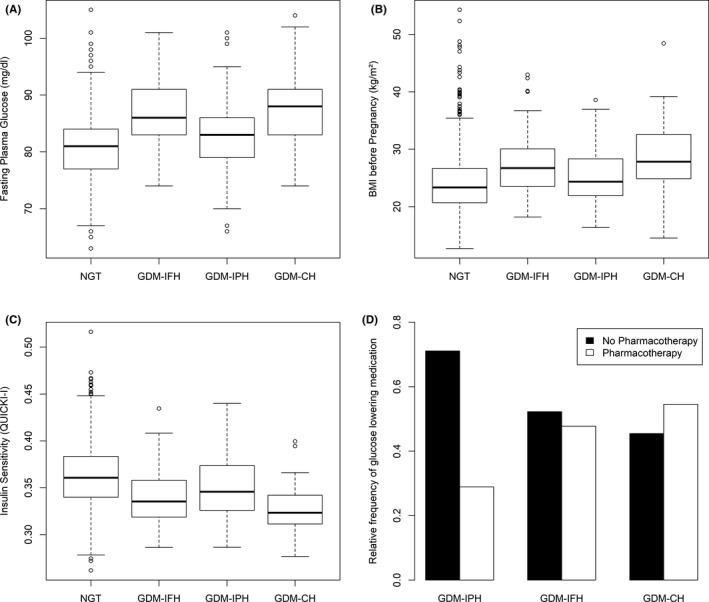
Box–whisker plots representing the difference between the investigated groups in fasting plasma glucose at early pregnancy (A), BMI before pregnancy (B), insulin sensitivity (C) and relative frequencies of patients with GDM who received glucose‐lowering medications presented as bar plots (D)

As provided in Table 1 all GDM subgroups showed impairments in glucometabolic parameters already at early gestation with higher FPG, HbA1c as well as a higher degree of insulin resistance as compared to NGT controls. However, there were also distinct differences observed between the studied groups. Especially the GDM‐CH subgroup showed worse metabolic profile, with higher insulin resistance and compensatory increased insulin release. Both GDM‐IFH and GDM‐CH had impaired β‐cell function as compared to NGT controls. As visualized in Figure 1, the GDM‐IPH subgroup showed significantly lower pregestational BMI as compared to GDM‐IFH, whereas triglycerides were increased as compared to NGT controls. Of note, the need of glucose‐lowering medication was higher in either GDM‐IFH (47.8%, OR = 2.25, 95% CI 1.15‐4.45, *P* = .019) as well as GDM‐CH (54.5%, OR = 2.95, 95% CI 1.39‐6.38, *P* = .005) as compared to GDM‐IPH (28.9%) and comparable results were observed after adjustment for maternal age and BMI (Figure 1). There were no major differences in parameters of main interest between patients with isolated post‐load hyperglycaemia at 1 hour vs 2 hour during the OGTT (Table S1). Moreover, our results did not change when 31 patients with early OGTT (before 24 weeks of gestation) were excluded (data not shown). A sensitivity analysis including only women with Caucasian origin is provided as supplemental material (Table S2), but did not change our basic conclusions.

**TABLE 1:** Characteristics of the study sample at study entry

	NGT (n = 893)	GDM‐IFH (n = 67)	GDM‐IPH (n = 83)	GDM‐CH (n = 44)
Age (y)	31.5 ± 5.8	32.0 ± 5.5	33.1 ± 5.3*	33.3 ± 6.0
Parity (≥1)	541 (60.6)	50 (74.6)	55 (66.3)	28 (63.6)
GDM in previous pregnancy	52 (5.8)	16 (23.9)*	20 (24.1)*	14 (31.8)*
Ethnicity (non‐Caucasian)	184 (20.6)	20 (29.9)	18 (21.7)	18 (40.9)*
BMI, before pregnancy (kg/m^2^)	24.3 ± 5.2	27.7 ± 5.5*	25.4 ± 4.8^†^	28.6 ± 6.3*, ^§^
Family history of diabetes	386 (43.2)	32 (47.8)	56 (67.5)*	31 (70.5)*
Multiple pregnancy	107 (12.0)	8 (11.9)	9 (10.8)	1 (2.3)
Triglycerides, early pregnancy (mg/dL)	114 ± 44.2	126 ± 43.2	139 ± 55.1*	144 ± 47.6*
Total cholesterol, early pregnancy (mg/dL)	188 ± 35.0	187 ± 32.6	196 ± 37.5	185 ± 36.2
LDL cholesterol, early pregnancy (mg/dL)	94.1 ± 27.9	99.7 ± 27.1	96.1 ± 29.4	93.8 ± 28.0
HDL cholesterol, early pregnancy (mg/dL)	71.1 ± 16.1	62.3 ± 12.5*	71.9 ± 16.5^†^	62.3 ± 12.3*, ^§^
FPG, early pregnancy (mg/dL)	80.6 ± 5.8	86.5 ± 5.7*	82.5 ± 7.0*, ^†^	87.7 ± 7.4*, ^§^
HbA1c, early pregnancy (%)	4.95 ± 0.29	5.09 ± 0.25*	5.07 ± 0.29*	5.24 ± 0.31*, ^§^
HbA1c, early pregnancy (mmol/mol)	30.6 ± 3.2	32.1 ± 2.8*	31.9 ± 3.2*	33.7 ± 3.4*, ^§^
Fasting insulin, early pregnancy (µU/mL)	7.5 (5.3‐10.7)	11.3 (7.2‐15.7)*	9.5 (6.2‐12.9)*	13.6 (10.0‐18.1)*, ^§^
Fasting C‐Peptide, early pregnancy (ng/mL)	1.50 (1.2‐1.9)	1.9 (1.5‐2.5)*	1.8 (1.4‐2.2)*	2.3 (2.1‐2.9)*, ^†^, ^§^
HOMA‐IR, early pregnancy (dimensionless)	1.46 (1.00‐2.16)	2.37 (1.53‐3.40)*	1.93 (1.17‐2.87)*	3.06 (2.08‐4.06)*, ^§^
QUICKI‐I, early pregnancy (dimensionless) × 10^2^	36.2 ± 3.4	34.1 ± 3.1*	35.2 ± 3.4*	32.7 ± 2.6*, ^§^
QUICKI‐CP, early pregnancy (dimensionless) × 10^2^	48.0 ± 3.8	45.3 ± 3.5*	46.5 ± 3.8*	43.4 ± 2.9*, ^§^
IGI, early pregnancy (ng/mg)	2.04 ± 0.75	2.37 ± 0.83*	2.26 ± 0.76*	2.77 ± 0.70*, ^†^, ^§^
DI, early pregnancy (ng/mg (µU/mL)^−1^) × 10^2^	24.7 (20.7‐31.0)	21.1 (17.6‐25.7)*	22.9 (19.2‐28.8)	19.2 (16.1‐23.7)*, ^§^

Note

Data are mean ± SD or median (IQR) and count (%) for women remaining normal glucose tolerant (NGT) vs patients developing gestational diabetes (GDM) with impaired fasting plasma glucose (IFH), impaired post‐load glucose (IPH) or both (CH).

Abbreviations: BMI, body mass index; DI, disposition index; FPG, fasting plasma glucose; HbA1c, glycated haemoglobin; HOMA‐IR, homeostasis model assessment of insulin resistance; IGI, insulinogenic index; QUICKI‐CP, quantitative insulin sensitivity check index from C‐peptide; QUICKI‐I, quantitative insulin sensitivity check index from insulin.

* *P* < .05 vs NGT.

^†^ *P* < .05 vs GDM‐IFH.

^§^ *P* < .05 vs GDM‐IPH.

Further analyses, which included only patients who developed GDM, revealed that insulin sensitivity (OR = 0.83, 95% CI 0.74‐0.92, *P* < .001 for the increase of 1 unit of QUICKI × 10^2^) and β‐cell function at early gestation (OR = 0.93, 95% CI 0.89‐0.97, *P* = .003 for the increase of 1 unit of DI × 10^2^) were inversely associated with the requirement of pharmacotherapy. Out of all three glucose values assessed during the OGTT, only fasting glucose was significantly associated with the need of glucose‐lowering medication (OR = 1.06, 95% CI 1.03‐1.10, *P* < .001 for the increase of 1 mg/dL), whereas glucose levels at 1 hour (*P* = .698) and 2 hours (*P* = .407) after oral glucose load did not reach significance. An optimal cut‐off value of 84 mg/dL fasting glucose showed a sensitivity of 92.5% (and therefore an acceptable negative predictive value of 86.7%), although the specificity was modest (34.2%). Detailed information about glucose values assessed during the diagnostic OGTT and use of glucose‐lowering medications are provided in Table 2. There was no difference between the groups regarding gestational weight gain until end of pregnancy (NGT: 15.4 ± 6.4; GDM‐IFH: 14.9 ± 7.4; GDM‐IPH: 13.8 ± 5.8; GDM‐CH: 14.0 ± 9.1, *P* = .506).

**TABLE 2:** Glucose values assessed during the diagnostic OGTT and pharmacotherapy

	NGT (n = 893)	GDM‐IFH (n = 67)	GDM‐IPH (n = 83)	GDM‐CH (n = 44)
OGTT Glucose 0 min (mg/dL)	78.6 ± 6.6	96.9 ± 5.7*	81.8 ± 6.5*, ^†^	99.4 ± 6.4*, ^§^
OGTT Glucose 60 min (mg/dL)	124.3 ± 26.8	145.5 ± 22.9*	187.0 ± 17.3*, ^†^	206.8 ± 21.7*, ^†^, ^§^
OGTT Glucose 120 min (mg/dL)	101.5 ± 20.2	110.3 ± 18.9*	145.1 ± 29.8*, ^†^	153.2 ± 25.6*, ^†^
Pharmacotherapy (insulin or metformin)	–	32 (47.8)	24 (28.9)*	24 (54.5)^§^
Pharmacotherapy (insulin)	–	23 (34.3)	18 (21.7)	16 (36.4)
Pharmacotherapy (metformin)	–	8 (11.9)	5 (6.0)	5 (11.4)
Pharmacotherapy (insulin and metformin)	–	1 (1.5)	1 (1.2)	3 (6.8)

* *P* < .05 vs NGT.

^†^ *P* < .05 vs GDM‐IFH.

^§^ *P* < .05 vs GDM‐IPH.

Regarding offspring data, we did not identify differences in foetal biometry and pregnancy outcomes between the studied subgroups (Table 3). No significant associations between birth weight percentiles and insulin sensitivity (QUICKI: rho = −0.10, *P* = .229) or β‐cell function (DI: rho = −0.11, *P* = .166) were observed in treated women with GDM.

**TABLE 3:** Foetal biometry and pregnancy outcomes (multiple pregnancies and cases with missing pregnancy outcome data are excluded)

	NGT (n = 763)	GDM‐IFH (n = 56)	GDM‐IPH (n = 70)	GDM‐CH (n = 43)	*P*‐value
Induction of foetal lung maturation	51 (6.6)	2 (3.5)	7 (10.0)	2 (4.8)	.484
Caesarean section	337 (43.8)	28 (49.1)	28 (39.4)	21 (48.8)	.654
Vacuum extraction	38 (5.0)	3 (5.3)	4 (5.6)	0 (0.0)	.219
Neonatal intensive care unit	40 (5.3)	5 (8.8)	4 (5.7)	1 (2.4)	.574
GAD (wks)	38.7 ± 1.8	38.6 ± 1.4	38.3 ± 1.6	38.4 ± 1.5	.332
Preterm delivery (<37 wks)	64 (8.3)	3 (5.3)	8 (11.3)	4 (9.3)	.574
Birth weight (percentile)	57 ± 28	64 ± 26	59 ± 30	56 ± 32	.372
LGA	108 (14.2)	9 (16.1)	13 (18.6)	9 (20.9)	.529

Note

Data are mean ± SD or median (IQR) and count (%) for women remaining normal glucose tolerant (NGT) vs patients developing gestational diabetes (GDM) with impaired fasting plasma glucose (IFH), impaired post‐load glucose (IPH) or both (CH).

Abbreviations: GAD, gestational age at delivery; LGA, large for gestational age offspring.

## 4 DISCUSSION

This study aimed to assess characteristics of GDM subtypes classified by fasting and/or post‐load hyperglycaemia during the diagnostic OGTT at mid‐gestation. We found that women affected by both (elevated fasting and post‐load hyperglycaemia) showed adverse metabolic profiles including a higher degree of insulin resistance at beginning of pregnancy as compared to NGT women or to those with isolated abnormalities of glucose metabolism. Of note, distinct differences were also observed between women with GDM who showed isolated impairments in either fasting or post‐load glucose. In particular, the GDM‐IPH subgroup had lower BMI and required glucose‐lowering medications less often as compared to GDM‐IFH and GDM‐CH. Moreover, the GDM‐CH subgroup showed increased proportions of GDM‐associated risk factors like family history of diabetes and were more likely to be of non‐Caucasian origin. However, our results remained unchanged when we accounted for ethnicity in a sensitivity analysis.

The possible phenotypical heterogeneity of GDM was also addressed in previous research, although most of these studies used different approaches to categorize GDM subtypes.^2^, ^20^, ^21^ Most recently, Benhalima et al^21^ classified GDM cases into physiologic phenotypes according to their degree of insulin sensitivity and found that women with GDM and higher degree of insulin resistance had higher BMI as well as increased FPG concentrations and showed an adverse lipid profile at early pregnancy, possibly associated with a greater risk of pregnancy complications. In another study, Powe et al^2^ found that GDM subtypes with predominant defects in insulin sensitivity had a higher risk of GDM‐associated adverse pregnancy outcomes as well as larger infants as compared to patients with predominant defects in insulin secretion or women with NGT. In addition, Liu et al^20^ found that GDM patients with insulin resistance, and particularly those with additional defects in β‐cell function, showed the most unfavourable metabolic profile and had the greatest risk for GDM‐related complications. While the concept of GDM phenotyping by use of advanced glucometabolic indices aiming to provide a classification on the basis of insulin action and secretion is plausible, it has the major disadvantage that the assessment and calculation of these parameters is rather complex and often requires multiple measurements of glucose, insulin and C‐peptide. Moreover, no reference values are available, especially for pregnancy. Therefore, we followed a different, more practical approach, by classifying GDM subtypes according to increased fasting and/or increased post‐load OGTT glucose concentrations. Studies on non‐pregnant humans suggested that impaired fasting and impaired post‐load hyperglycaemia during an OGTT characterize different metabolic entities.^5^ In line with these observations, we found that mothers with isolated fasting glucose tended to have an unfavourable metabolic profile as compared to those with isolated post‐load hyperglycaemia. Of note, women classified as GDM‐IFH as well as GDM‐CH received glucose‐lowering medications more than twice as often as women with isolated post‐load hyperglycaemia. This may be explained by impaired β‐cell dysfunction in GDM‐IFH and GDM‐CH subgroups, as indicated by our study already at beginning of pregnancy. In line with these results, we identified an inverse association between insulin sensitivity and β‐cell function at early gestation with later requirement of pharmacotherapy in patients with GDM. It is noteworthy to mention that out of all three glucose values assessed during the OGTT fasting glucose was the only significant predictor for the need of glucose‐lowering medication. While the promising properties of fasting glucose were also addressed in one of our previous studies,^22^ the overall accuracy of a specific cut‐off value was rather modest. An optimal threshold of 84 mg/dL showed an acceptable sensitivity and negative predictive value but lacked in specificity.

We observed no differences in either the risk for LGA or other obstetric outcomes. This is in contrast with previous research suggesting a significant association between FPG levels and LGA development or neonatal adiposity.^23^, ^24^ Also, another retrospective report from the Kaiser Permanent Southern California Medical Care Program suggested that the magnitude and significance of adverse pregnancy outcomes differed by various combinations of abnormal OGTT glucose values: while women with elevated post‐load glucose concentrations were at higher risk for hypertension, preterm delivery or infants with hyperbilirubinaemia, women with elevated fasting glucose showed increased risk for having LGA offspring.^25^ In addition, a recent meta‐analysis identified stronger associations of LGA (or pregnancy outcomes) with fasting glucose than with post‐load glucose.^26^ Therefore, the majority of published pregnancy outcome data corroborates our hypothesis that classification of GDM subtypes according to fasting and post‐load hyperglycaemia is of clinical importance and should be considered in clinical routine to provide individual treatment strategies. However, the heterogeneous results between our study and previous studies regarding pregnancy outcomes may be explained by local differences in diagnostic algorithms as well as therapeutic regimes.

One limitation of this study is that blood examinations, and especially assessment of insulin sensitivity and β‐cell function, were only performed once at early gestation by use of fasting blood examinations. Although we were able to show that fasting parameters of insulin sensitivity and β‐cell function were significantly associated with parameters assessed during the 1h‐FSIGT in an independent validation cohort, we are aware that fasting parameters may have restricted reliability to describe the underlying physiological mechanisms. However, this study design was chosen in order to identify possible risk constellations already at the beginning of pregnancy in a large cohort of study participants. The large sample size is another relevant advantage of this work.

In summary, we observed that GDM subtypes classified by either fasting and/or post‐load hyperglycaemia during the diagnostic OGTT at mid‐gestation showed distinct metabolic characteristics already at beginning of pregnancy. Mothers with GDM and fasting hyperglycaemia, and especially those with elevated fasting and post‐load glucose levels, had an unfavourable metabolic phenotype with higher degree of insulin resistance and were more likely to receive glucose‐lowering medications. While it is still a matter of discussion how to distinguish GDM subtypes with lower or higher risk for adverse pregnancy outcomes, we conclude that the categorization based on abnormal OGTT values provides a good and practicable basis for clinical risk stratification and future research.

## CONFLICTS OF INTEREST

The authors declare that they have no competing interests.

## AUTHOR CONTRIBUTIONS

CSG conceived the study. Data assessment and patient recruitment were performed by IR, GK, JB, DE, TL and CSG. Calculations and data interpretation were performed by CSG and AT. Statistical analysis was performed by CSG. CSG prepared tables and figures. The manuscript was written by CSG and GK. AT, IR, DE, JB, IH, EAH, FG and TL critically revised the manuscript. All authors reviewed and edited the final draft of the manuscript. The authors have nothing to disclose. CSG and GK serve as guarantors and accept full responsibility for the work and/or the conduct of the study, had access to the data and controlled the decision to publish.

## Supporting information

Supplementary Material
