# Supplementary material for: Characteristics of gestational diabetes subtypes classified by oral glucose tolerance test values
Source: Eur J Clin Invest. 2021 Jun 13;51(9):e13628. doi: 10.1111/eci.13628 (PMC8459269; doi:10.1111/eci.13628)
Supplement: Supplementary file 1 — Supplementary Material [file ECI-51-e13628-s001.docx]

**Appendix A. Supplementary data**

**Figure S1:** Flow-chart representing included and excluded patients


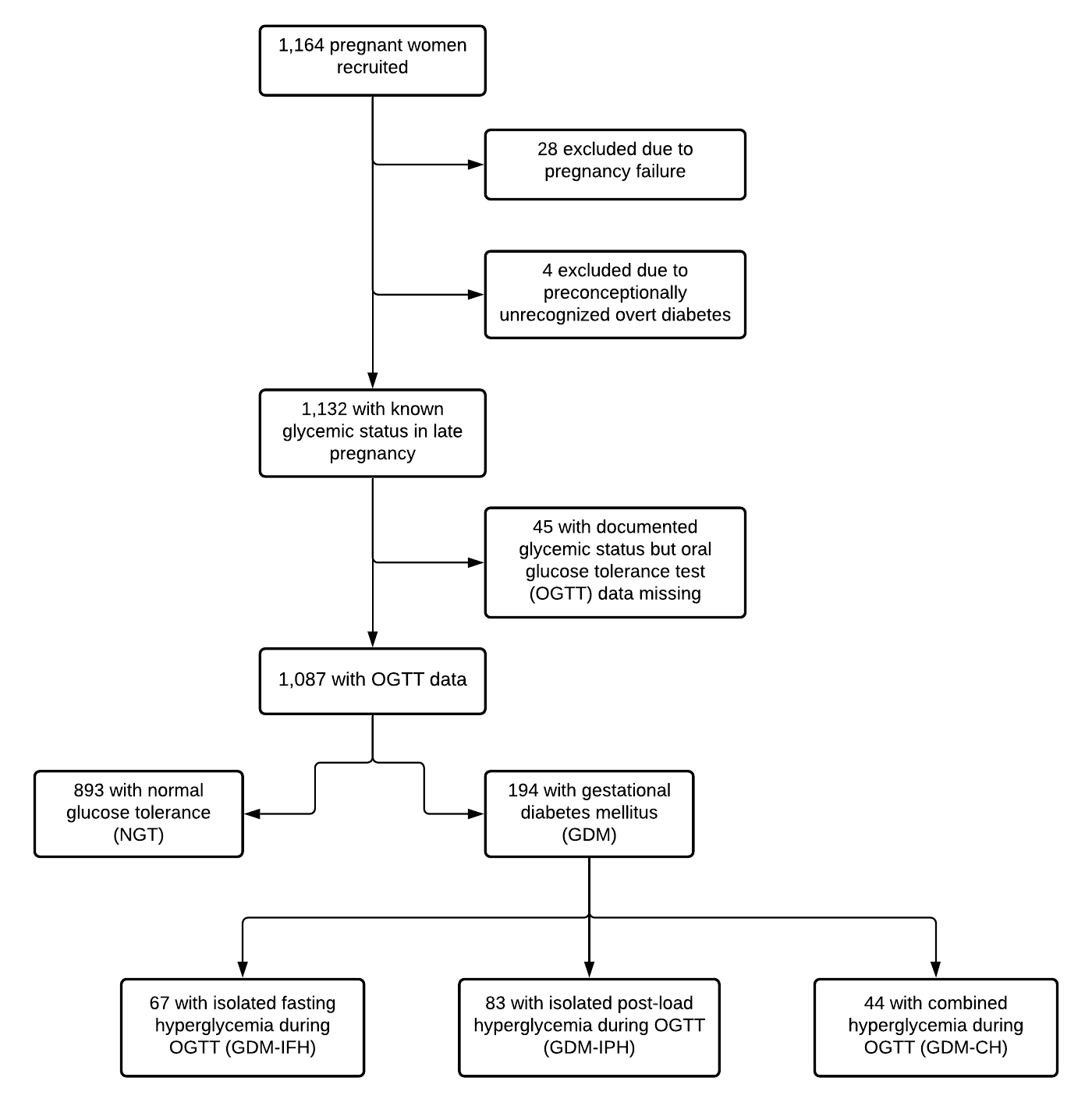


**Figure S2:** Results of a validation cohort showing the relationship between self-measured and technician-measured BMI (A), insulin sensitivity (QUICKI-CP) and the insulin sensitivity calculated from the 1h-FSIGT(CSI) (B) as well as the association between the disposition index (DI) derived from fasting parameters and the 1h-FSIGT (C). For illustration purposes data transformations were performed: LN(X+1) for QUICKI-CP, CSI and 1h-FSIGT derived DI; -1/(X+10) for DI derived from fasting parameters.

**
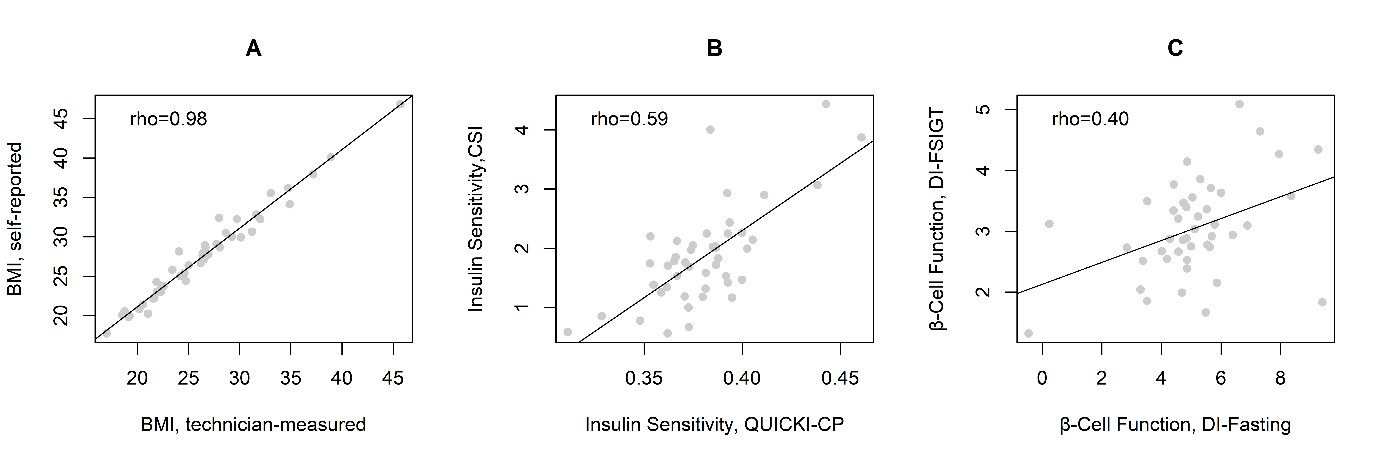
**

**Table S1:** Comparison of patients with GDM and isolated post-load hyperglycemia at 1h (GDM-PLH-1h) and at 2h (GDM-PLH-2h) during the OGTT

|  | **GDM-PLH-1h** | **GDM-PLH-2h** | **p-value** |
| --- | --- | --- | --- |
|  | **(n=46)** | **(n=20)** |  |
| Age (years) | 32.9±4.9 | 33.5±6.4 | 0.750 |
| BMI, before pregnancy (kg/m^2^) | 26.6±5.1 | 23.4±4.2 | 0.011 |
| FPG, early pregnancy (mg/dl) | 82.6±5.7 | 81.8±9.0 | 0.730 |
| HbA1c, early pregnancy (%) | 5.03±0.30 | 5.07±0.21 | 0.519 |
| Fasting insulin, early pregnancy (µU/ml) | 10.0 (7.3-13.2) | 7.9 (4.9-9.9) | 0.094 |
| Fasting C-Peptide, early pregnancy (ng/ml) | 1.8 (1.5-2.2) | 1.5 (1.3-2.2) | 0.231 |
| HOMA-IR, early pregnancy (dimensionless) | 2.06 (1.50-2.88) | 1.64 (0.99-1.99) | 0.142 |
| QUICKI-I, early pregnancy (dimensionless) × 10² | 34.7±3.0 | 36.2±4.2 | 0.170 |
| QUICKI-CP, early pregnancy (dimensionless) × 10² | 46.2±3.4 | 47.2±4.4 | 0.385 |
| IGI, early pregnancy (ng/mg) | 2.31±0.70 | 2.21±0.93 | 0.684 |
| DI, early pregnancy (ng mg^-1^ (µU/ml)^-1^) × 10² | 22.7 (19.5-25.6) | 27.3 (21.3-32.5) | 0.094 |
| Pharmacotherapy (Insulin and/or Metformin) | 15 (32.6) | 5 (25.0) | 0.537 |
| Birth weight offspring (percentile) | 61.7±31.0 | 61.0±25.6 | 0.901 |

Data are mean±SD or median (IQR) and count (%). BMI, body mass index; FPG, fasting plasma glucose; HbA1c, glycated hemoglobin; HOMA-IR, homeostasis model assessment of insulin resistance; QUICKI-I, quantitative insulin sensitivity check index from insulin; QUICKI-CP, quantitative insulin sensitivity check index from C-peptide; IGI, insulinogenic index; DI, disposition index

**Table S2:** Sensitivity analysis of glucometabolic parameters in early gestation after excluding women with non-Caucasian origin

|  | **NGT** | **GDM-IFH** | **GDM-IPH** | **GDM-CH** |
| --- | --- | --- | --- | --- |
|  | **(n=709)** | **(n=47)** | **(n=65)** | **(n=26)** |
| Age (years) | 31.7±5.9 | 32.1±5.6 | 33.5±5.1 | 32.9±6.7 |
| FPG, early pregnancy (mg/dl) | 80.5±5.8 | 85.9±5.6* | 82.4±7.2† | 86.5±6.8*§ |
| HbA1c, early pregnancy (mmol/mol) | 30.4±3.2 | 32.0±2.4* | 31.4±3.2 | 33.4±3.2*§ |
| Fasting insulin, early pregnancy (µU/ml) | 7.4 (5.2-10.7) | 11.4 (7.9-16.7)* | 8.9 (5.6-12.6) | 12.9 (9.6-16.5)*§ |
| Fasting C-Peptide, early pregnancy (ng/ml) | 1.5 (1.2-1.9) | 1.9 (1.8-2.6)* | 1.7 (1.4-2.2) | 2.3 (2.0-2.7)*§ |
| HOMA-IR, early pregnancy (dimensionless) | 1.45 (0.98-2.16) | 2.40 (1.72-3.34)* | 1.85 (1.14-2.76) | 2.73 (1.99-3.49)*§ |
| QUICKI-I, early pregnancy (dimensionless) × 10² | 36.3±3.4 | 33.9±3.1* | 35.4±3.5 | 33.1±3.0*§ |
| QUICKI-CP, early pregnancy (dimensionless) × 10² | 48.0±3.8 | 44.8±3.4* | 46.7±3.9* | 43.8±3.2*§ |
| IGI, early pregnancy (ng/mg) | 2.04±0.74 | 2.53±0.81* | 2.25±0.81 | 2.74±0.75*§ |
| DI, early pregnancy (ng mg^-1^ (µU/ml)^-1^) × 10² | 25.2 (21.0-31.6) | 21.1 (17.7-26.6)* | 24.3 (19.9-29.1) | 19.7 (17.3-24.2)* |

Data are mean±SD or median (IQR) and count (%) for women remaining normal glucose tolerant (NGT) vs. patients developing gestational diabetes (GDM) with impaired fasting plasma glucose (IFH), impaired post-load glucose (IPH) or both (CH). BMI, body mass index; FPG, fasting plasma glucose; HbA1c, glycated hemoglobin; HOMA-IR, homeostasis model assessment of insulin resistance; QUICKI-I, quantitative insulin sensitivity check index from insulin; QUICKI-CP, quantitative insulin sensitivity check index from C-peptide; IGI, insulinogenic index; DI, disposition index

* p<0.05 vs. NGT

† p<0.05 vs. GDM-IFH

§ p<0.05 vs. GDM-IPH
